# Having pity on our victims to save ourselves: Compassion reduces self-critical emotions and self-blame about past harmful behavior among those who highly identify with their past self

**DOI:** 10.1371/journal.pone.0223945

**Published:** 2019-12-12

**Authors:** Ernst Willem Meerholz, Russell Spears, Kai Epstude

**Affiliations:** Department of Social Psychology, University of Groningen, Groningen, The Netherlands; Universite de Lille, FRANCE

## Abstract

Previous research has shown that people often separate the present self from past selves. Applying knowledge gained from intergroup research to the interpersonal domain, we argue that the degree to which people identify with their past self (self-identification) influences their reaction when recalling a past event during which they harmed another person. Because they feel close to their past self, we expected this to be threatening for high self-identifiers, and expected them to be motivated to avoid self-critical emotions and blame. Using four meta-analyses, conducted on a set of seven experimental studies, we investigated four ways in which high self-identifiers can distance themselves from the event: by feeling compassion, by taking a third-person rather than first-person perspective, by emphasizing ways in which their present self is different to their past self, and by disidentifying with the past self altogether. We found the strongest interaction effects for compassion: whereas a compassion manipulation increased self-critical emotions and self-blame about the past event for low self-identifiers, it decreased them for high self-identifiers. We argue that this occurs because the other-focused nature of compassion allows high self-identifiers subtly to shift the focus away from their harmful behavior. Our concept of past self-identification had stronger effects than a measure of self-continuity beliefs. It also correlated only moderately with the latter, suggesting they are distinct concepts. Our findings suggest that, ironically, the most effective way to protect the self against reminders of an undesirable past, may be to have compassion for our victims.

## Introduction

What happens when we are confronted with our own past bad behavior, behavior that harmed someone else? Do we see it as water under the bridge, or does it threaten our perception of ourselves as a good person? And if we do feel threatened by the ghosts from our past, how do we deal with them? In this paper, we investigate whether the way we feel about a memory of our past negative behavior is influenced by the degree to which we identify with the person we were back then. We believe that evoking such memories is not threatening for low (personal) identifiers (people who feel they have little in common with the person they were when the event took place). After all, they may well think they are a very different person nowadays. In contrast, we do expect the information to be threatening for high identifiers, because they will likely feel they are still very much the same person. As a result, the past transgression therefore reflects more on their current self. Using four meta-analyses, conducted on a set of seven experimental studies, we investigate four potential ways in which high self-identifiers can subsequently deal with these identity threats: a. by experiencing compassion for the person they harmed; b. by taking a third- rather than first-person perspective when thinking about the situation; c. by focusing on ways in which they are different today compared to when the events took place; and d. by actively disidentifying from their past self during the course of the research.

### The past self

An underlying assumption in our studies is that people differentiate between their present self and past selves. This view is not without detractors in psychology. For example, James [1] argued that people need to believe that the self is stable over time in order to have a sense of personal identity. Early research on the link between identity and autobiographical memory seemed to support James' idea. These studies showed that when people assess to what extent they have changed, they rely on implicit theories about the stability of personal characteristics and feelings [2]. If you were to ask a person whether their attitude about a particular issue has changed, they would first appraise their current view of this issue. After this, they need to determine what their previous position on this topic was. This is where their implicit theories come into play: if they believe that people are stable over time, this makes them more inclined to think that their past attitude was similar to their current attitude [3]. Ross [2] summarized several studies on this topic and concluded that although variation exists, most people tend to hold the implicit belief that their attitudes and attributes are indeed relatively stable over time. He concluded that, as a result, people tend to overestimate how consistent they actually are. The finding that people tend to believe in stability and the resulting perceptions of personal consistency, do not seem to be in line with our assumption of past-present self-differentiation.

However, more recent research paints a different picture. Wilson and Ross [3] describe that newer research has shown that people actually *do* frequently report shifts in identity over time. One way in which this is evident, is that people tend to rate their past selves more negatively than their present self. A key reason for this is the fact that people are motivated to see their current self positively [4,5]. In fact, Wilson and Ross argue that people can actively construct shifts in their identity to distance themselves from unfavorable past selves. As they put it: "distant failures lose their power to taint and glories to flatter the present self" [3 p139]. In a sense, our past selves can be cast aside. Several studies have shown that people tend to perceive, appraise and judge the past self in much the same way as they perceive other people (e.g [6]). Recent research has even found evidence for this at the neural level [7]. However, this work also shows that we do not *entirely* distance ourselves from our past incarnations: specifically, people process information about their past selves similarly to how they process information about close others. Taken together, this seems to suggest that we might be protective of our past self in the way we might be protective of a friend, but not to the same degree as we are about the present self.

In short, the existing literature supports our assumption that people differentiate between the past and present selves. People also vary in terms of the implicit theories they hold about personal stability and consistency, and their past experiences may influence to what extent they perceive shifts in their identity. Someone with few or not very severe 'distant failures' may not perceive as many identity shifts across time as someone with many, because there is less of a reason for them to create a schism. Memory and identity mutually influence each other in this regard [3].

### Identifying with the past self

The fact that people differentiate between past and present selves, and differ in the degree to which they do, is important. When confronted with past harmful behavior, we believe that the degree to which people perceive their present self to be similar to their past self (what we refer to as self-identification), functions similarly to identification at the group level. Research in the social identity tradition suggests that a confrontation with past harmful behavior by the ingroup constitutes an identity threat for high identifiers [8]. This is because the group is very important to them, and because people are motivated to have a positive perception of the groups they belong to. Low identifiers, in contrast, are much more open to self-critical group emotions, such as group-based guilt, shame and anger. Research has shown that high identifiers can deal with these threats in various ways. For instance, they may downplay the harm that was done, deny responsibility, or blame the victims [9]. However, there are situations where this may simply not be feasible.

We think this is particularly likely in interpersonal situations, where there are simply fewer 'means of escape'. Intergroup situations offer ways to distance oneself from the harmful group behavior that are simply unavailable in interpersonal situations. For instance, people are often not personally, not directly, or at least not individually responsible for the harmful behavior of the group: this gives an individual the (plausible) option to deny their involvement or responsibility and thus distance themselves from group actions. They may also create subgroups within their ingroup and blame 'a few bad apples' for the harmful behavior. None of these options are available when it comes to harm inflicted by the individual self on another individual. How can a highly self-identified individual who is directly responsible for harming someone else deal with an identity threat of this nature? We believe one thing they can do is, counterintuitively, to experience compassion for the person they harmed.

### Feeling compassion

In a previous line of intergroup research [10] (for a precursor to this work also see [11]), we investigated whether compassion can reduce self-critical emotions among highly identified members of a group who are confronted with past harmful behavior by their group towards an outgroup. This prediction seems counterintuitive at first. Several studies have shown that compassion has positive effects for both interpersonal and intergroup relations. For instance, it is associated with an increased positive attitude towards another group [12]. Compassion is defined as “being moved by another’s suffering and wanting to help” [13 p289]. It is a highly other-focused emotion and it is this strong focus on the suffering of the other to which many of its positive effects are attributed (e.g. [14,15]). Importantly, however, compassion does not imply responsibility for the observed suffering [16].

Self-critical emotions like group-based guilt, shame and anger are, in contrast to compassion, all inherently self-focused. This is where the other-focus so strongly associated with compassion comes in handy. We proposed that compassion might allow high identifiers to shift the focus away from the harmful behavior of their ingroup and thereby reduce these emotions. In other words, compassion allows high identifiers to focus on feeling sorry for the outgroup, rather than feeling self-critical emotions regarding their ingroup's behavior. For low identifiers, we predicted that compassion would either have no effects, or lead to an increase of self-critical emotions. This is simply because low identifiers, unlike high identifiers, have little reason to be defensive.

In a meta-analysis of five studies about the Dutch colonial history in Indonesia using Dutch participants, we found support for this idea [10]. A compassion manipulation (an instruction to think about the suffering of the outgroup) reduced self-critical emotions among high identifiers, as well as the willingness to support reparation attempts and the degree to which participants felt responsible. However, the manipulation did not significantly affect low identifiers. In a sixth study, using the same context, we found evidence for our proposed mechanism [10]. Participants were randomly assigned to either a self-focus or other-focus condition (we showed them pictures of either the ingroup or the outgroup). We found the same negative effects for high identifiers in the other-focus condition, and positive effects for low identifiers, suggesting that it is the general shift in focus from self to other that drives our compassion effects. These findings are particularly noteworthy given the subtle nature of the mechanism (a simple shift in focus).

In the present set of studies, we asked participants to recall and describe an event that happened five to ten years ago where they harmed another person, and used the same compassion manipulation as in our intergroup research. Because we expect that identification with the past self functions like group identification in contexts where people are confronted with past harm, we derived our predictions from our intergroup findings. We predicted that compassion would reduce guilt, shame, anger and regret about the past event among high self-identifiers. Based on our findings for responsibility perceptions in our intergroup studies, we also predicted that compassion would reduce the degree to which high identifiers blame themselves for the event and that it would increase the degree to which they blamed the person they harmed. For low identifiers, we expected to find null effects, or positive effects.

### Taking a third-person perspective

Compassion is of course not the only way in which the focus can be shifted away from the self, and perhaps not the most direct or straightforward. We were also interested in testing whether describing the past event in either a first- or third-person perspective would influence how participants felt about this event. As we discussed above, people already have a natural tendency to perceive past selves in a somewhat more distant manner, specifically, in the same way they would look at a close other. However, we wanted to see whether this distance can be enhanced by varying participants' perspectives.

There is existing evidence that a third-person perspective is indeed associated with increased subjective distance from an event. In early research, Nigro and Neisser [17] found that memories that were older, less vivid, and that contained less emotional and more objective information about the situation, tended to be visually recalled more from a third-person perspective than a first-person perspective [3]. Furthermore, Fergusson [18] found that people who wrote about negative events that happened to them from a third-person perspective reported lower levels of distress about these events four weeks after the writing task, compared to participants who wrote about such events from a first-person perspective. Participants in the third-person group even paid fewer visits to a health center up to fifty days later, compared to participants in the first-person group.

Additional evidence stems from more contemporary research on self-distancing. For example, in a line of research by Kross and Ayduk [19], participants recalled negative events, and analyzed their feelings about this event from a ‘self-distanced’ perspective (visualizing the event from the external perspective of a fly on the wall) or a ‘self-immersed’ perspective (reliving the experience from their own, original perspective). Across several studies, involving events triggering various emotions (e.g. sadness, anxiety, guilt), the authors found that participants re-experience their original negative emotions to a lesser extent when they take a self-distanced perspective. Furthermore, Kross et al. [20] showed that reflecting on a past failure using third-person pronouns was associated with better stress regulation and decreased levels of distress compared to using first-person pronouns. A systematic review by Wallace-Hadrill and Kamboj [21] also concluded that taking on a third-person perspective in studies in which participants recalled upsetting memories was generally associated with lower negative affect.

The research cited above seems to suggest that adopting a third-person perspective while recalling a negative past event will always result in the experience of less negative affect, because it creates more psychological distance from the event. However, Libby and Eibach [22] offer a different account, which suggests that the effects of adopting a third-person perspective may be more nuanced, and depend on other variables. Specifically, they argue that visualizing events from a first-person perspective causes people to construe actions and events in a bottom-up style, meaning that they primarily focus on concrete features of the event, such as the emotions they experienced in the moment. In contrast, they argue that a third-person perspective triggers a top-down process in which events are construed in a more abstract manner. For example, people may consider the causes and consequences, or the broader meaning of the event to a larger degree. Crucially, they argue that this more abstract construal is not *necessarily* associated with a reduction in negative affect, in contrast with the abovementioned self-distancing account. Rather, they argue that how people make sense of an event from this more abstract third-person perspective, depends on “the knowledge structures and motivations that define the self-concept” [23 p1169]. In support of this, they found that self-esteem moderated the effect of perspective on feelings of shame. In line with research on self-distancing, imagining failure from a third-person perspective led to decreased feelings of shame among participants with high self-esteem. However, it led to an *increase* among participants with low self-esteem [23]. This is likely because the more abstract nature of the third-person perspective caused these low self-esteem participants to perceive the failure as a general reflection of the self, whereas high self-esteem participants considered it as atypical against the backdrop of their otherwise positive self. In sum, this line of research highlights the role that the self-concept and its associated motivations play in explaining and predicting the effects of adopting a third-person perspective.

This brings us to the role that identification with the past self may play. With regards to motivation, it is important to consider whether high self-identifiers would *want* to distance themselves from the past self. The fact that they are highly identified with the past self seems to imply that they would not want to differentiate themselves from this past self. We believe that the answer to this is two-sided. One the one hand, we believe that high self-identifiers do not want to distance themselves from their past self. Creating a schism between the past self and present self would suggest that they could not be highly identified with their past self anymore, and runs counter to the self-protective motivation that is characteristic of high identifiers. On the other hand, we do think that they want to distance themselves from the negative past *event* in any way possible. Adopting a third-person perspective would give them the opportunity to do so. Consistent with the work by Libby et al. [22], we do not expect that taking a third-person perspective inherently forces people to create distance between their past and present self. Rather, it gives them an opportunity to create distance: whether they use this opportunity to distance themselves from the event or from a past self may depend, in part, on their motivations.

With regards to high identifiers, it seems clear that they have a strong motivation to reduce self-critical emotions when recalling an instance where they harmed someone, because they have a strong desire to cast the past self in a positive light. Therefore, they should make the most of the opportunity to distance themselves from the event. We thus predicted that, like compassion, adopting a third-person perspective will significantly reduce self-critical emotions and self-blame, and increase other-blame for high identifiers. For low identifiers, we did not state clear predictions. We considered that we might find effects in the same direction, but that these could be either weak or non-existent. The reason for this is that these people are already weakly identified with the past self and should not have a high motivation to further distance themselves. An additional distancing mechanism is therefore unlikely to exert a strong effect.

### Emphasizing differences

Thirdly, we tested whether simply getting people to focus on ways in which they are different versus similar to their past self would affect their emotions. We asked participants either to list three ways in which they are similar, or three ways in which they are different compared to the person they were five to ten years ago, prior to recalling the event. Previous research has shown that focusing participants on similarities between the present and past self causes them to assimilate perceptions of the present self to the past self, whereas focusing them on differences causes them to contrast the current self away from the past self [24]. This suggests that focusing participants on differences should allow them to create distance between the past and present self. We wanted to see whether this indeed occurs, specifically whether it would subsequently translate into a reduction in self-critical emotions.

Here, we again re-affirm the point we made in the discussion of the perspective manipulation: we do not expect high self-identifiers to want to create distance between the past and present self. To be clear, participants were free to list whichever differences or similarities they chose. In other words, they were not forced or pressured to create distance between the past and present self. Whether they wanted to list differences that indicated this type of distance (e.g. "I am a friendlier person nowadays") or differences that are much more mundane (e.g. "I no longer enjoy pasta") was entirely up to them. We expected that giving high identifiers the opportunity to list ways in which they are currently different should again give them an opportunity to shift the focus away from the (past) self. Therefore, we predicted that for high identifiers the salient difference-manipulation would also reduce self-critical emotions and self-blame, and increase other-blame. For low identifiers, we expected a null effect or a positive effect (we did not state a clear prediction). These people very possibly already feel quite different to the person they were when the event took place. For them focusing on ways in which they were different could have no significant effect, but might also increase this even further and as a result create more distance between the past and present self. This could make them even more open to self-critical emotions.

### Distancing through disidentification

Finally, we ran an exploratory analysis to see whether identification with the past self was affected by the emotions evoked during the experiment. We included both a pre- and a post-measure of self-identification in all seven studies and analyzed the interaction effects between the pre-measure of identification and the measured self-critical emotions on the post-measure of identification. We did not have specific predictions for this analysis. Because low identifiers are known to be more open to self-critical emotions and already feel distinct from their past self, it is reasonable to assume that self-reported guilt, shame, anger or regret should not alter their self-identification that much. For high identifiers, an effect seemed more likely to occur. As we mentioned above, we know that people actively create shifts in their identity so that past failures can no longer taint them. This is of course not what a high self-identifier would ideally want, but perhaps the only viable strategy 'if all else fails'. Could it be that this would happen over the course of our experiment? That recalling the past event and the emotions this would evoke would cause them to identify less with the past self at the end of the experiment? We tested this possibility.

## Method

We present the results of four meta-analyses, using data from a set of seven studies that we conducted on this topic. There are two major advantages to using a meta-analysis approach. Firstly, it gives us a more reliable estimate of the true effect size in the population. This is particularly relevant when individual studies have low statistical power. Although the samples we garnered for each individual study were likely larger than that of the average study in psychology, the effect sizes we found turned out to be small. The sample sizes were therefore not sufficient to find reliable effects within the individual studies. However, the meta-analysis approach gives us overall effect sizes that are "functionally equivalent to the test of a hypothesis in a single study with high power" [25 p563], thereby counteracting this problem. A second advantage to this approach is that it allows us to include all the studies that we conducted in this line of research, including those that 'failed', so that none end up in the proverbial file-drawer. This is obviously important, given that the psychological literature suffers from bias due to the fact that unsuccessful studies are often not published [26]. Although the use of meta-analysis for a single set of studies (rather than an entire literature) is still relatively novel, various authors have argued for the advantages of this approach (e.g. [25–28]) and/or applied it already (e.g. [29,30]).

### Design

All studies included in the manuscript were approved by the Ethical Committee Psychology at the University of Groningen. All participants submitted written consent prior to participating in the included studies. The set-up of the seven studies was the same, but we varied the manipulations that we used from study to study. We used the compassion manipulation in Studies 1, 2, 3 and 5, the perspective manipulation in Studies 4, 5, and 6, and the list (of similarities or differences) manipulation in Study 2 and 3. These manipulations are described in detail below. Pre- and post-measures of self-identification were included in all seven studies. It should be noted that Study 7 contained a focus manipulation. We do not report findings for this manipulation because it was only used in one of the seven studies (and given that we found very small effect sizes, we did not deem it viable to analyze this variable). Further research, using bigger samples, is needed to check the validity of this effect.

In each study, participants filled out a pre-measure of identification with the past self. They were then asked to recall an event that happened 5 to 10 years ago during which their behavior, in one way or another, harmed somebody else. We asked them: "What was the situation, and what was it that you did? Reflect on this a bit before continuing." On the next page, participants were then asked to briefly describe this event in an empty textbox presented on-screen. We subsequently measured our dependent variables. The dependent variables that we included in the meta-analyses were the four self-critical emotions guilt, shame, anger and regret, and a measure of self- and victim-blame. We also measured other variables for exploratory purposes. Information about these will be provided by the authors upon request.

### Participants

Participants were recruited on the online Amazon Mechanical Turk platform and received $0.50 in exchange for their participation. The studies were created using Qualtrics. Table 1 provides an overview of the sample size per study and the demographic characteristics of our participants, as well as an overview of the design of each study. Participants were randomly assigned to experimental conditions in all studies.

**Table 1:** Methodological characteristics of the seven studies included in the meta-analysis.

Study	Design	IVs & conditions	N	Participant characteristics
1	Single factor	-Compassion manipulation: yes or no	139	-Age range: 18–74 *-M* = 34.31, *SD* = 12.71 -97 women, 41 men, 1 unknown
2	2 x 2	-Compassion manipulation: yes or no -Salient difference manipulation: similarities or differences	291	-Age range: 18–73 *-M* = 35.44, *SD* = 12.20 -193 women, 98 men
3	2 x 2	-Compassion manipulation: yes or no -Salient difference manipulation: similarities or differences	292	-Age range: 18–77 *-M* = 34.94, *SD* = 12.71 -193 women, 99 men
4	Single factor	-Perspective manipulation: 1st or 3rd person	146	-Age range: 19–66 *-M* = 34.84, *SD* = 12.95 -88 women, 58 men
5	2 x 2	-Perspective manipulation: 1st or 3rd person -Compassion manipulation: yes or no	267	-Age range: 18–69 *-M* = 35.28, *SD* = 11.20 -175 women, 92 men
6	Single factor	-Perspective manipulation: 1st or 3rd person	306	-Age range: 18–72 *-M* = 34.21, *SD* = 12.56 -193 women, 111 men, 2 unknown
7	Single factor	-Focus manipulation: self or other	301	-Age range: 18–67 *-M* = 34.21, *SD* = 11.55 -210 women, 91 men

We investigated the open-ended descriptions of the events we asked participants to recall. Most participants followed our instructions properly, describing events that ranged from unfortunate accidents, to offending others, to disappointing loved ones, to extramarital affairs and even criminal behavior. However, there were some exceptions to this. Firstly, some participants did not describe an event clearly. This included people who simply did not try (one participant wrote "3", for instance), to participants who might have made a serious attempt but whose description was so unclear that it was impossible to be certain of this (for example, one participant wrote "I forgot the cookie"), to people who indicated that they never harmed someone (e.g. "I really haven't harmed anyone in the last 5–10 years—sorry :/"). We removed these cases from the dataset.

However, there were also more complicated cases. Some participants described actions where it was not entirely clear who they harmed. For instance, one participant wrote "I got a DUI 10 years ago." In cases like this, we cannot be sure whether the participant was thinking of a specific person. This could be problematic for our study, because all our manipulations and measures refer to a specific other individual that was harmed (e.g. "I feel guilty about what I did to the person I harmed"). However, because our instructions clearly state that participants were supposed to think of an event where they harmed another person, we deemed this unlikely. Because of this, we did not remove participants with answers like this.

In some cases it seemed clear that participants simply misread our instructions, but other cases were more ambiguous. For instance, one person wrote: "my feelings were hurt by one of my friends so I quit speaking to her for a while." On the surface, this might suggest that our participant misinterpreted our instruction. However, this participant did indicate feeling moderate levels of guilt (presumably about ostracising their friend). Equally complex were the answers of participants who seemed to have interpreted our instruction to remember an event where they harmed someone else literally. That is, in terms of physical harm. One participant, for instance, described: "This redneck kept grabbing my girlfriend at the bar. So I threw him on the ground and punched him repeatedly." Another participant described "finally" beating up his bully. It is of course possible that these participants felt guilty about their behavior, but it could also be that they misinterpreted our instruction.

We decided to be conservative and not remove people who seemed to describe episodes where someone else harmed them, or people who seemed to physically retaliate against someone else. Only cases where the description was incomprehensible or did not appear to be serious were removed. Every dataset also contained several incomplete cases (almost always participants who backed out very soon after launching the survey). These were also removed. Table 2 gives an overview of the number of total cases in each of the datasets, the number of incomplete and uninterpretable cases that were removed, and the final number of cases that was used in the analyses.

**Table 2:** An overview of the total cases in each dataset, as well as the number of incomplete and uninterpretable cases that were removed, and the size of the remaining samples used in the analyses.

Study	Total cases in dataset	Incomplete cases	Uninterpretable cases	N used in analyses
1	194	42	13	139
2	365	58	16	291
3	374	67	15	292
4	203	47	10	146
5	352	74	11	267
6	388	75	7	306
7	393	75	17	301

### Materials and procedure

#### Identification with past self

We measured identification with the past self with two items in Study 1 (α = .88) and 2 (α = .89). We adapted the two items forming the 'self-stereotyping' subscale of the 14-item in-group identification scale developed by Leach et al. [31] ("I have a lot in common with the person I was 5 to 10 years ago", "I am similar to the person I was 5 to 10 years ago"). Participants were asked to rate these items from 1 (*not at all*) to 7 (*very much*). This measure was presented at the beginning of our study. We showed participants the same measure again near the end of the study, to create a post-measure as well, for which the Cronbach's alpha values varied between α = .88 to α = .94. In the other five studies we added one additional item to the pre-measure (not to the post-measure), "I identify with the person I was 5 to 10 years ago." The Cronbach's alpha for the pre-measure in these five studies ranged from α = .90 to α = .93. The analyses we present using identification are based on the two-item measure in Studies 1 and 2 and the three-item measure in all other studies.

#### Compassion manipulation

We manipulated compassion in Studies 1, 2, 3 and 5 by either giving participants a short instruction to think about the suffering of the person they harmed (intended to increase the salience of compassion) or no instruction at all. This instruction was presented after participants completed the recall task. As part of our compassion manipulation, we also added two items (‘I feel compassion for the person I harmed’, ‘I feel sympathy for the person I harmed’. We added these items to further increase the salience of compassion in the compassion condition. In the four studies in which we manipulated compassion, we varied the order in which participants were shown these two items and our measure of guilt, our most prototypical self-critical emotion. Participants in the compassion condition first answered the questions about compassion and then about guilt; participants in the control condition first answered the questions about guilt and then about compassion. Order manipulations of this kind have been used previously to make the independent variable of interest more salient (e.g. the salience of education in Study 3 by Kuppens, Easterbrook, Spears & Manstead [32]). For the sake of consistency, we also included the compassion items in our other studies, but without varying the order in which they were presented.

#### Perspective manipulation

The perspective manipulation that we used in Studies 4, 5 and 6 was integrated into the recall task. We added an extra paragraph in which we told participants that in order to help them reimagine this event, we ask them to use a specific technique that has previously been found to be a good way to relive past events. They were then told to think about and briefly describe the event in either the first person or in the third person. On the next page, which included the empty textbox, we emphasized this once more.

It should be noted that in our analysis of the effects of the perspective manipulation, we excluded 8 additional participants (3 in Study 4, 1 in Study 5, and 3 in Study 6) because they did not follow our perspective manipulation adequately (e.g. writing in the third person when they were instructed to write in the first person). To be clear, these 8 participants were *not* excluded from the other analyses conducted on these datasets (regarding the compassion manipulation for Study 5, and regarding disidentification for all three studies). This was because participants did describe a recalled event appropriately, and we thus did not deem it justified to exclude them.

#### Salient difference manipulation

The salient difference (versus similarity) to the past self-manipulation that was employed in Studies 2 and 3 was presented prior to the recall task. We told participants that we are interested in the ways in which people perceive themselves throughout their lifetime. We then asked them to take a moment to think about the person they were 5 to 10 years ago, and to then list three ways in which they think they are currently the same as, or different to (depending on the condition), the person they were at that time. Detailed descriptions of all three manipulations are available upon request.

#### Guilt

Guilt was measured with three items, 'I feel guilty about what I did to the person I harmed', 'I easily feel guilty about the bad outcomes for the person I harmed', and 'My behavior towards the person I harmed easily makes me feel guilty'. We used these items in our previous intergroup research on this topic [10] and changed them so that they fit an interpersonal context. These items were originally adapted from earlier research [11,33,34]. The Cronbach's alpha ranged from α = .91 to α = .95 in the seven studies.

#### Shame

Shame was measured with three items. We used the same items we used for the guilt scale, but with the word 'ashamed' replacing 'guilty' (alpha between α = .94 and α = .97).

#### Anger

We measured anger with two items, 'When I think about the way I treated the person I harmed, I feel furious' and 'I feel angry towards myself for the way I treated the person I harmed'. These items were again adapted from our previous intergroup studies, and were originally based on a scale used by Iyer, Schmader, and Lickel [35] (alpha between α = .88 and α = .91).

#### Regret

Regret was measured with two items (alpha between α = .92 and α = .95), 'I regret what I did to the person I harmed' and 'I feel remorse for what I did to the person I harmed'. All the emotion measures described above used the same scale, ranging from 1 (not at all) to 7 (very much).

#### Self-blame

Self-blame was assessed with one item. Participants were asked to what extent they blamed themselves for the situation they recalled. They could move a slider from 0 to 100 to the percentage that they felt applied. We used this slider format because we thought participants may find it more natural to think of blame in terms of percentages (e.g. “I am 75% to blame, the other person 25%), rather than in terms of a 7-point scale.

#### Other-blame

Other-blame was measured in the same way. Participants were now asked to what extent they believed the person they harmed was to blame for the situation.

## Results

Below, we present the results of the four meta-analyses we conducted. In each meta-analysis, we looked at the two way interaction between our (centered) pre-measure of identification and the relevant manipulation or measure. In Studies 2, 3 and 5, we used multiple manipulations at the same time. We do not present three-way interactions, both because we did not predict specific three-way interactions and because our samples were not large enough to test these effects reliably.

**Fig 1:**
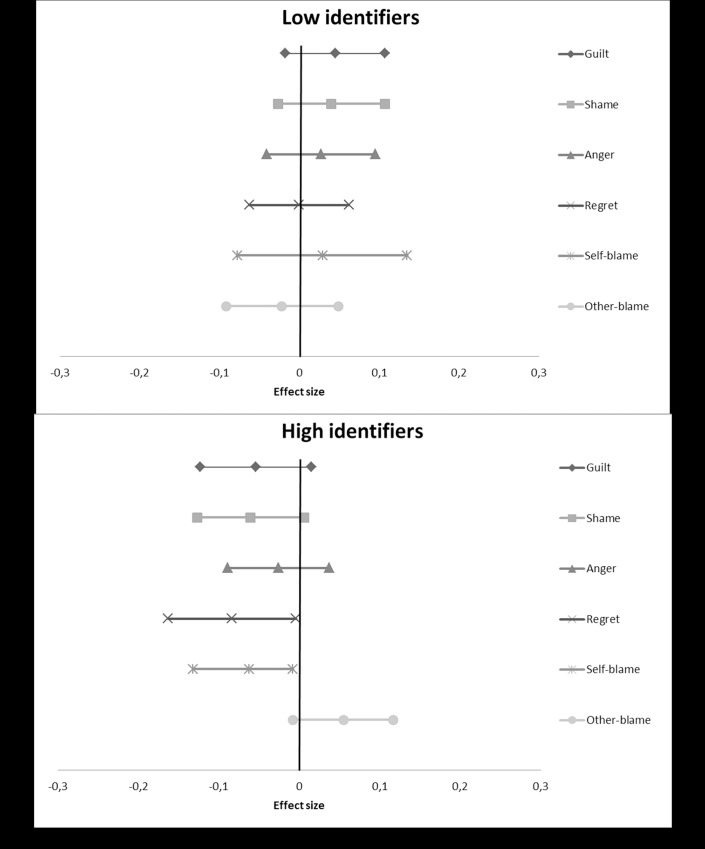
A forest plot showing the overall meta-analytic simple main effects of our compassion manipulation on the dependent variables (based on four studies). Effect sizes are provided along with their confidence intervals. A positive value indicates that participants in the compassion condition scored higher than participants in the control condition, a negative value that they scored lower.

**Fig 2:**
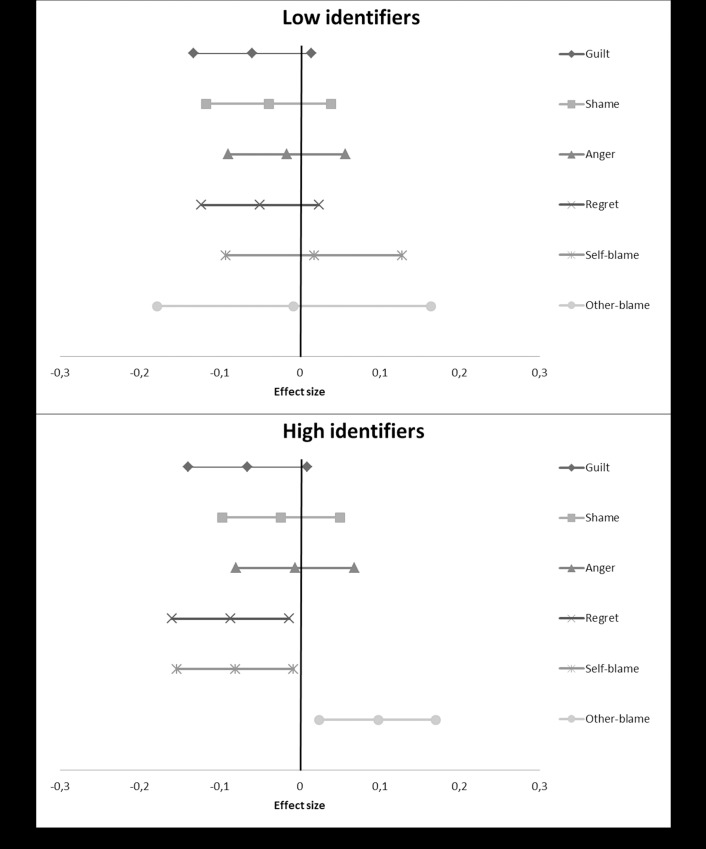
A forest plot showing the overall meta-analytic simple main effects of our perspective manipulation on the dependent variables (based on three studies). Effect sizes are provided along with their confidence intervals. A positive value indicates that participants in the third person-perspective condition scored higher than participants in the first person-perspective condition, a negative value that they scored lower.

**Fig 3:**
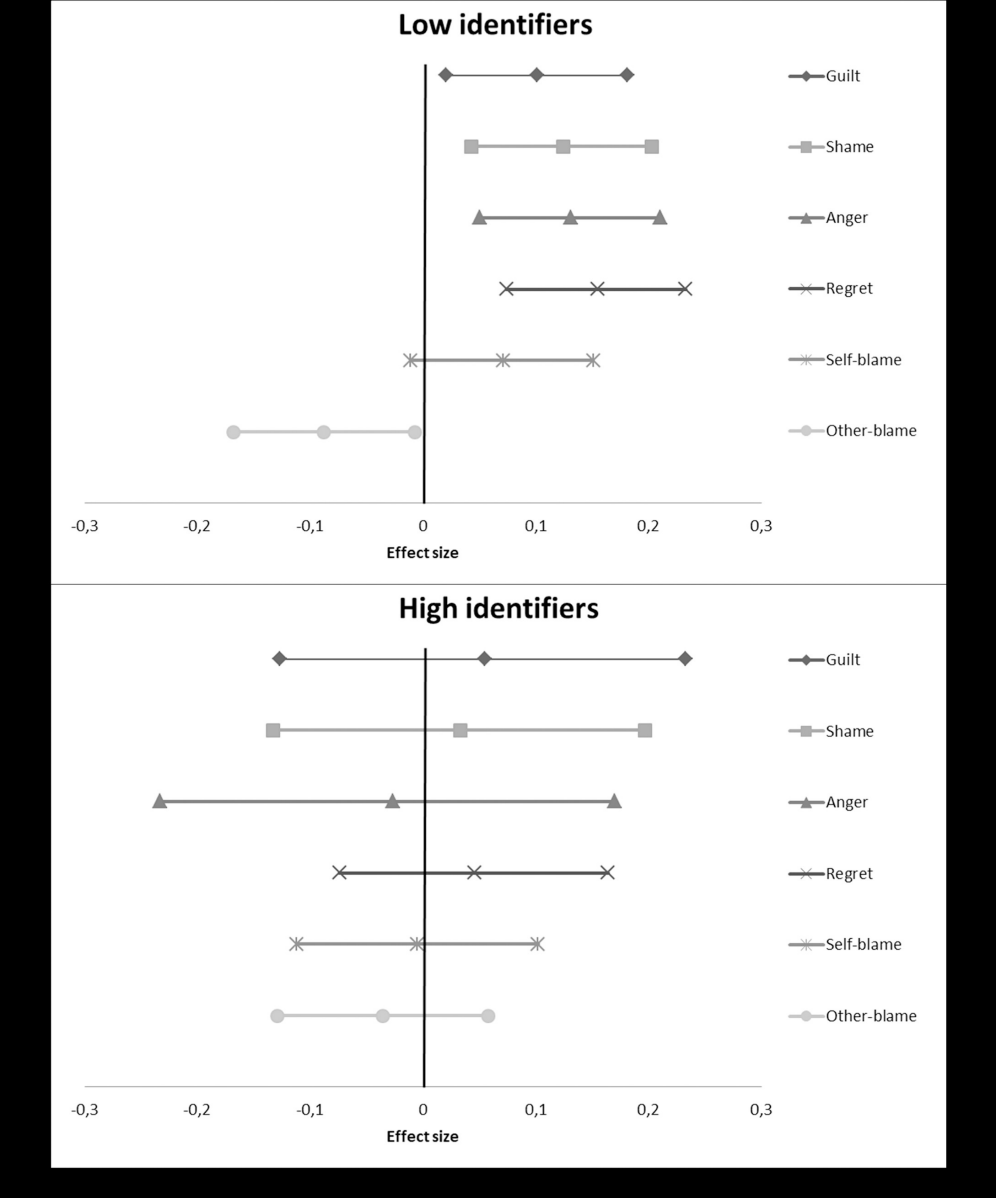
A forest plot showing the overall meta-analytic simple main effects of our salient difference-manipulation on the dependent variables (based on two studies). Effect sizes are provided along with their confidence intervals. A positive value indicates that participants in the difference-focused condition scored higher than participants in the similarities-focused condition, a negative value that they scored lower.

**Fig 4:**
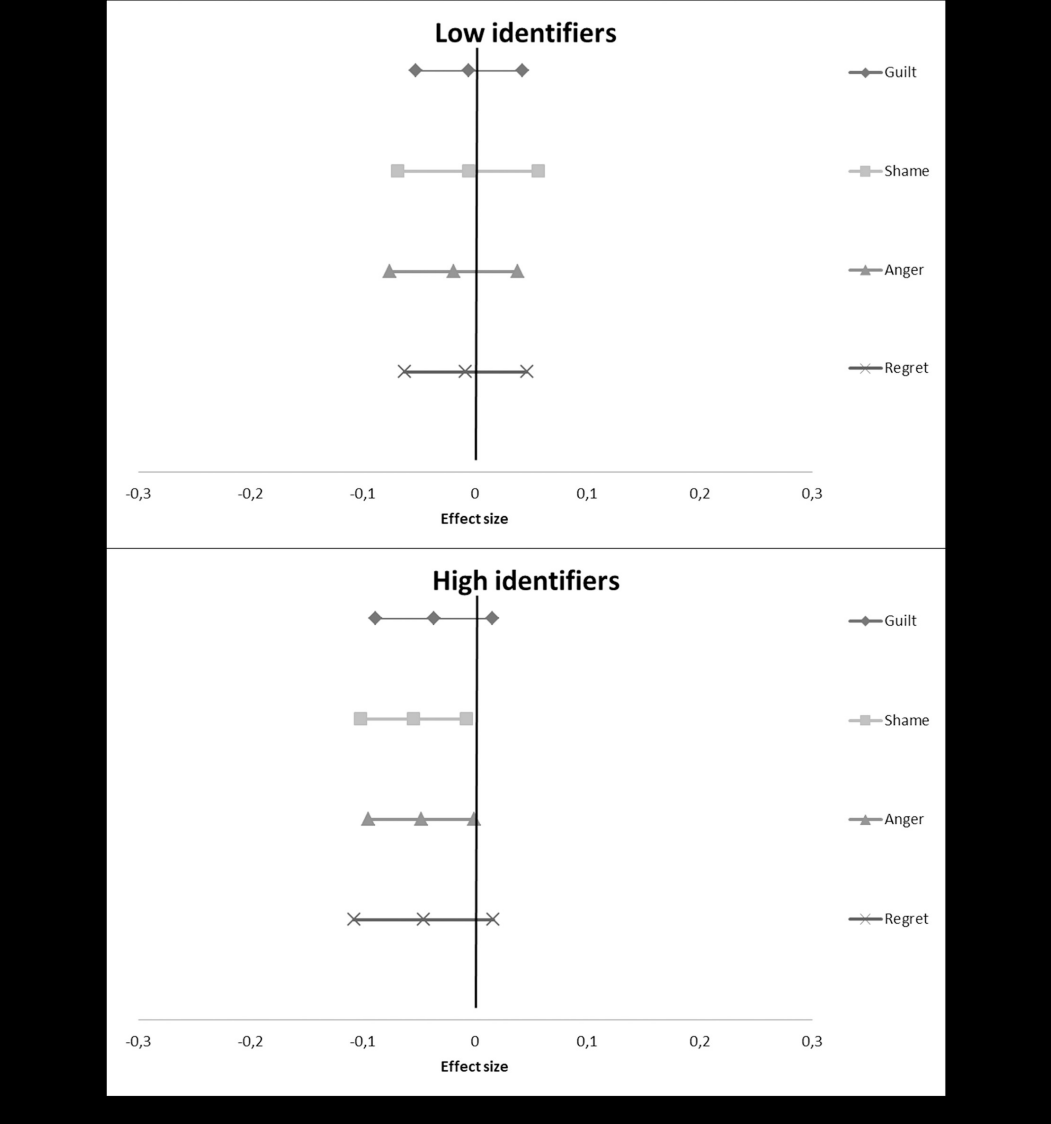
A forest plot showing the overall meta-analytic effects (based on seven studies) of the interaction between our pre-measure of identification on the one hand, and guilt, shame, anger and regret on the other. The dependent variable was our post-measure of identification. Effect sizes are provided along with their confidence intervals. A positive value indicates that participants scored higher on the post-measure of identification than on the pre-measure, a negative value that they scored lower, as a function of the emotion.

We used the PROCESS macro for SPSS [36] to calculate the results for each individual study. Subsequently, we took the t-values for both the overall interaction effect as well as the simple main effects, and converted these into r-values using the program D-Stat [37]. We then entered these converted r-values into MetaLight [38], to calculate overall effect sizes along with confidence intervals, to assess significance, as well as to calculate a Q-statistic. This statistic tests for heterogeneity, whether the variation in the effects found in the individual studies is larger than would be expected by chance. A significant Q-statistic indicates that this is the case and is thus problematic (i.e. there may be moderator variables operating). Although our studies were highly similar methodologically, we made a conservative choice and used random effects models rather than fixed effects models. We provide forest plots showing the overall meta-analytic simple main effects of our compassion manipulation (Fig 1), perspective manipulation (Fig 2), and salient difference-manipulation (Fig 3), as well as the analysis of the effect on disidentification as a result of the emotions evoked during the study (Fig 4). More detailed forest plots, showing the effects found in each individual study alongside the overall meta-analysis effects, are available in an electronic appendix upon request.

### Compassion manipulation

To test whether the compassion manipulation reduced self-critical emotions and self-blame, and increases victim blaming among high identifiers, we calculated overall effect sizes for the interactions between our manipulation and pre-measure of identification, based on Studies 1, 2, 3 and 5. It should be noted that we also ran additional analyses in order to assess a potential confound. Specifically, we asked two independent judges to rate the events participants recalled in terms of the severity of harm that was inflicted on the victim. We then tested whether self-identification affected this severity, and found that it did have a significant negative effect. We subsequently tested whether it could account for our findings regarding the compassion, perspective, and salient difference manipulations, and re-ran our original analyses while controlling for severity (as well as the interaction between severity and the manipulation). We found that doing so did not change our findings compared to our original analyses, as reported here, in a noteworthy manner. Full information can be found in S1 Appendix.

#### Guilt

The overall effect size for the interaction term across the four studies, *r* = -0.07, was significant, 95% CI (-0.13, -0.06) and heterogeneity was not a problem, Q(3) = 0.93, *p*>.250. Fig 1 shows forest plots for the simple effects for low and high identifiers, providing information about the nature of the interaction. The forest plot contains the overall effect size for each of the dependent variables. A positive effect here indicates that participants in the compassion condition reported more guilt compared to participants in the control condition. As Fig 1 shows, the compassion manipulation reduced guilt among high identifiers, in line with our hypothesis. The simple effect for high identifiers was nearly significant, *r* = -0.06, 95% CI (-0.12, 0.01), Q(3) = 3.62, *p*>.250. In contrast, the simple effect for low identifiers showed a positive trend, *r* = 0.05, 95% CI (-0.02, 0.11), Q(3) = 2.82, *p*>.250. The compassion manipulation thus reduced feelings of guilt among high identifiers.

#### Shame

The overall interaction effect for shame was also significant, *r* = -0.07, 95% CI (-0.13, -0.01), Q(3) = 1.74, *p*>.250. As can be seen in Fig 1, we see the exact same pattern, again in line with our hypothesis. There was a negative near significant effect for high identifiers, *r* = -0.06, 95% CI (-0.13, 0.01), Q(3) = 3.37, *p*>.250, and a slightly weaker, positive near-significant effect for low identifiers, *r* = 0.04, 95% CI (-0.03, 0.11), Q(3) = 3.38, *p*>.250.

#### Anger

In contrast to guilt and shame, the overall interaction for anger was not significant, *r* = -0.04, 95% CI (-0.10, 0.02), Q(3) = 1.62, p>.250. The simple effects (Fig 1) do follow the same pattern, but it is weaker. Both the effects for high identifiers, *r* = -0.03, 95% CI (-0.09, 0.04), Q(3) = 2.65, p>.250, and low identifiers, *r* = 0.03, 95% CI (-0.04, 0.10), Q(3) = 3.50, p>.250 were not significant.

#### Regret

The overall interaction for regret was nearly significant, *r* = -0.06, 95% CI (-0.12, 0.01), Q(3) = 0.38, p>.250. Fig 1 shows that compassion significantly reduces regret among high identifiers, *r* = -0.08, 95% CI (-0.16, -0.00), Q(3) = 4.75, *p* = .191. For low identifiers, rather than a positive pattern, we now observe a null effect, *r* = -0.001, 95% CI (-0.06, 0.06), Q(3) = 3.00, p>.250.

#### Self-blame

For self-blame, the overall interaction effect was significant, *r* = -0.07, 95% CI (-0.14, -0.00), Q(3) = 3.69, p>.250. Fig 1 shows that high identifiers in the compassion condition blamed themselves to a lesser extent for the situation they recalled, this effect was nearly significant, *r* = -0.06, 95% CI (-0.13, 0.01), Q(3) = 3.71, p>.250. For low identifiers there appears to be a slightly positive overall effect, but this was not significant, *r* = 0.03, 95% CI (-0.08, 0.14), Q(3) = 7.59, *p* = .055.

#### Other-blame

The overall interaction for other-blame was nearly significant, *r* = -0.06, 95% CI (-0.01, 0.12), Q(3) = 1.74, p>.250. As Fig 1 shows, the simple effect for high identifiers was again nearly significant, *r* = 0.06, 95% CI (-0.01, 0.12), Q(3) = 2.44, p>.250. In line with our findings on self-blame, this effect is positive, indicating that high identifiers in the compassion condition blamed the other person to a greater extent for the situation they recalled. For low identifiers, the effect is again not significant, though it appears slightly negative (again, in line with the findings on self-blame), *r* = -0.02, 95% CI (-0.09, 0.05), Q(3) = 3.73, *p* = .292.

### Perspective manipulation

To test whether a switch from a first to a third person perspective reduces self-critical emotions and self-blame, and increases victim blaming among high identifiers, we calculated overall effect sizes for the interactions between our perspective manipulation and pre-measure of identification, based on Studies 4, 5 and 6. These are displayed in Fig 2.

#### Guilt

The interaction for guilt was not significant, *r* = -0.00, 95% CI (-0.09, 0.09), Q(2) = 2.65, p>.250. The overall effect size, shown in Fig 2, indicates that low- and high identifiers were affected similarly. There was a nearly significant simple effect for both high identifiers, *r* = -0.07, 95% CI (-0.14, 0.01), Q(2) = 2.04, p>.250, and low identifiers, *r* = -0.06, 95% CI (-0.13, 0.01), Q(2) = 0.99, p>.250. In other words, both high and low identifiers in the third person condition felt less guilt than participants in the first person condition.

#### Shame

The same pattern, albeit a lot weaker, appears for shame. The overall interaction was again not significant, *r* = -0.02, 95% CI (-0.08, 0.11), Q(2) = 3.10, p>.250. The negative simple effect for high identifiers was not significant, *r* = -0.02, 95% CI (-0.10, 0.05), Q(2) = 1.09, p>.250, nor was the negative simple effect for low identifiers, *r* = -0.04, 95% CI (-0.12, 0.04), Q(2) = 2.22, p>.250 (Fig 2).

#### Anger

The effect on anger was even weaker. There was no significant interaction, *r* = 0.01, 95% CI (-0.08, 0.11), Q(2) = 3.05, *p* = .218. As Fig 2 shows, the simple effects for high identifiers, *r* = -0.01, 95% CI (-0.08, 0.07), Q(2) = 1.60, p>.250 and low identifiers, *r* = -0.02, 95% CI (-0.09, 0.06), Q(2) = 1.65, p>.250 were not significant either.

#### Regret

For regret, we found stronger negative effects again. The overall interaction effect was again not significant, *r* = -0.02, 95% CI (-0.11, 0.07), Q(2) = 2.86, *p* = .239. Fig 2 shows that the effects for low identifiers and high identifiers again looked similar. The effect for high identifiers was significant, *r* = -0.09, 95% CI (-0.16, -0.01), Q(2) = 1.24, p>.250, and the effect for low identifiers was nearly significant, *r* = -0.05, 95% CI (-0.12, 0.02), Q(2) = 1.70, p>.250.

#### Self-blame

Although we found very similar results for low and high identifiers on the emotion measures, interesting differences emerged on the blame measures. Fig 2 shows the differences on self-blame. The overall interaction effect was nearly significant, *r* = -0.07, 95% CI (-0.16, 0.02), Q(2) = 2.91, *p* = .234. For high identifiers, the third-person perspective manipulation significantly decreased self-blame, *r* = -0.08, 95% CI (-0.15, -0.01), Q(2) = 1.08, p>.250. For low identifiers, we found a null effect, *r* = 0.02, 95% CI (-0.09, 0.13), Q(2) = 4.29, *p* = .117.

#### Other-blame

Unsurprisingly, the results on victim blaming complement those on self-blame. The overall interaction effect was again nearly significant, *r* = 0.07, 95% CI (-0.00, 0.14), Q(2) = 1.90, p>.250. As shown in Fig 2, high identifiers in the third person perspective-condition blamed the victim significantly more than those in the first person condition, *r* = 0.10, 95% CI (0.02, 0.17), Q(2) = 1.66, p>.250. We again found a null effect for low identifiers, *r* = -0.01, 95% CI (-0.18, 0.16), Q(2) = 10.50, *p* = .005. The Q-statistic was significant here, indicating heterogeneity.

### Salient difference manipulation

To test our hypothesis that high identifiers would feel less self-critical emotions and self-blame, and more other-blame, when given the opportunity to focus on ways in which they differ from their past self, we analyzed the interactions between our salient difference-manipulation and pre-measure of identification. This was based on the data of Studies 2 and 3. The overall effect sizes are displayed in Fig 3.

#### Guilt

The overall interaction for guilt was not significant, *r* = -0.03, 95% CI (-0.17, 0.10), Q(2) = 2.79, *p* = .095. As Fig 3 shows, there was a small positive, but not significant simple effect for high identifiers, *r* = 0.06, 95% CI (-0.13, 0.23), Q(2) = 5.02, *p* = .03. The Q-statistic was significant, indicating heterogeneity. The simple effect for low identifiers was significant however, *r* = 0.10, 95% CI (0.02, 0.18), Q(2) = 0.01, p>.250. Focusing participants on the ways in which they were different from (versus similar to) their past self did not significantly affect high identifiers, but increased feelings of guilt among low identifiers.

#### Shame

We found the same pattern for shame (Fig 3). The overall interaction effect was again not significant, *r* = -0.07, 95% CI (-0.21, 0.08), Q(2) = 3.26, *p* = .07. The simple effect for high identifiers was again slightly positive, but not close to being significant, *r* = 0.03, 95% CI (-0.13, 0.20), Q(2) = 4.19, *p* = .041. The Q-statistic was again significant. The simple effect for low identifiers was highly significant, *r* = -0.12, 95% CI (0.04, 0.20), Q(2) = 0.26, p>.250.

#### Anger

For anger, the overall interaction effect was not significant, *r* = -0.11, 95% CI (-0.25, 0.03), Q(1) = 3.14, *p* = .078. As can be seen in Fig 3, the simple effect for high identifiers was again not significant and suffered from heterogeneity, *r* = -0.03, 95% CI (-0.22, 0.17), Q(2) = 5.96, *p* = .015. In contrast to the other emotions, it was slightly negative here, however. The simple effect for low identifiers was again highly significant, *r* = 0.13, 95% CI (0.05, 0.21), Q(2) = 0.99, p>.250.

#### Regret

A very similar pattern was found on regret. The overall interaction was nearly significant, *r* = -0.08, 95% CI (-0.17, 0.01), Q(1) = 1.30, p>.250. Fig 3 shows a slightly positive, but not significant simple main effect for high identifiers, *r* = 0.05, 95% CI (-0.07, 0.16), Q(1) = 2.16, *p* = .140. For low identifiers, the simple effect was again highly significant, *r* = 0.16, 95% CI (0.07, 0.23), Q(2) = 0.02, p>.250.

#### Self-blame

The effects found for the salient difference-manipulation on emotions, also seem to translate to the blame measures. The overall interaction effect was again not significant, *r* = -0.05, 95% CI (-0.17, 0.06), Q(1) = 1.90, *p* = .168. As shown in Fig 3, we found a null effect for high identifiers, *r* = -0.001, 95% CI (-0.11, 0.10), Q(1) = 1.73, *p* = .188. For low identifiers, we found a nearly significant positive effect, *r* = 0.07, 95% CI (-0.01, 0.15), Q(1) = 0.40, p>.250.

#### Other-blame

The results for other-blame corresponded again (Fig 3). The overall interaction effect was again not significant, *r* = -0.04, 95% CI (-0.04, 0.12), Q(1) = 0.47, p>.250. For high identifiers, we found a slightly negative but non-significant effect, *r* = -0.04, 95% CI (-0.13, 0.06), Q(1) = 1.33, *p* = .250. For low identifiers, we found a significant negative effect, *r* = -0.09, 95% CI (-0.17, 0.01), Q(1) = 0.02, p>.250. Low identifiers thus blamed themselves more and the other person less, for the situation they recalled during the experiment.

### Disidentification effects

We explored whether participants would actively disidentify during the course of the experiment, as a result of the self-critical emotions that were triggered by the recall task. To test this, we calculated the interaction between our pre-measure and each of the emotion measures on the post-measure of identification, for each of the seven studies. The overall effect sizes are provided in Fig 4.

#### Guilt

Does the guilt evoked during the experiment cause participants to identify less with their former self at the experiment than at the beginning? We did not find a significant overall interaction effect, *r* = -0.24, 95% CI (-0.08, 0.03), Q(6) = 8.55, *p* = .200. Fig 4 shows the simple effects for low and high identifiers. A positive value here indicates that participants scored higher on the post-measure of identification than on the pre-measure, a negative value that they scored lower, as a function of the emotion they reported. We found a nearly significant negative simple effect for high identifiers in the predicted direction, *r* = -0.04, 95% CI (-0.09, 0.02), Q(6) = 7.26, p>.250. The guilt evoked during the experiment caused high identifiers to identify less with their past selves at the end of the study. For low identifiers, we found a null effect, *r* = -0.01, 95% CI (-0.05, 0.04), Q(6) = 5.19, p>.250.

#### Shame

For shame, the overall interaction was again not significant, *r* = -0.03, 95% CI (-0.09, 0.03), Q(6) = 10.60, *p* = .103. As Fig 4 shows, the simple main effect for high identifiers was significant, however, *r* = -0.06, 95% CI (-0.10, -0.01), Q(6) = 5.78, p>.250. We again found a null effect for low identifiers, *r* = -0.01, 95% CI (-0.07, 0.06), Q(6) = 10.10, *p* = .119.

#### Anger

The same pattern emerged for anger (Fig 4). The overall interaction effect was not significant, *r* = -0.02, 95% CI (-0.07, 0.03), Q(6) = 5.16, p>.250. The simple effect for high identifiers was again significant, *r* = -0.05, 95% CI (-0.09, -0.00), Q(6) = 4.42, p>.250. For low identifiers, the effect for anger was slightly more negative than that for guilt and shame, but not significant, *r* = -0.02, 95% CI (-0.08, 0.04), Q(6) = 8.58, p = .198.

#### Regret

Finally, the same pattern also emerged for regret (Fig 4). The overall effect was again not significant, *r* = -0.03, 95% CI (-0.08, 0.03), Q(6) = 8.12, *p* = .230. The simple effect for high identifiers was negative, albeit not significant, *r* = -0.05, 95% CI (-0.11, 0.02), Q(6) = 10.10, *p* = .119. For low identifiers we found a null effect, *r* = -0.01, 95% CI (-0.06, 0.05), Q(6) = 7.91, *p* = .245.

## Discussion

Previous research has shown that people differentiate between the person they are today and the person they used to be [3]. Past selves tend to be perceived in a more distant manner, in the same way that we perceive close others, like a friend [6,7]. People also vary in the extent to which they differentiate between the present and past selves. The purpose of this paper was to investigate how people react when they are confronted with their past harmful behavior. We argued that in this situation, the degree to which people identify with/feel similar to their past self (which we refer to as self-identification) should function in the same way as group identification does at the intergroup level. That is, whereas low identifiers (people who see themselves as very different today compared to in the past) should be open to feeling self-critical emotions such as guilt, shame, anger and regret about their past behavior, high identifiers (people who see themselves as very similar to their past self) are expected to react defensively because the confrontation with past negative behavior constitutes a threat to the (current) self-concept. Thus we expect high identifiers to want to avoid self-critical emotions, because they are highly motivated to retain a positive view of their past self, which is after all essentially also their current self.

Using a meta-analytic approach to our program of research on this topic, we looked at four possible ways in which high identifiers could reduce self-critical emotions and feelings of blame: a. By experiencing compassion for the person they harmed, b. By taking a third- rather than first-person perspective when thinking about the situation, c. By focusing on ways in which they are different today compared to when the events took place, and d. By actively disidentifying with their past self during the course of the experiment. Our results generally supported the compassion and perspective (a and b) hypotheses, but showed weak support for the salient-differences hypothesis (c). We did not have specific predictions regarding the disidentification strategy (d). Although we found consistent patterns, not all effects were significant, and effect sizes were small.

### Hypothesis 1: Compassion manipulation

Based on earlier intergroup research [10], we expected compassion to reduce self-critical emotions for high identifiers because its other-focused nature allows them to shift the focus away from their harmful behavior. Our results supported this idea. We found significant overall interaction effects on guilt and shame, and a nearly significant effect on regret. The effect on anger was not significant, but the pattern was similar. The simple effects were, with the exception of regret for high identifiers, not significant, but usually at least near-significant for high identifiers. In general, we found that compassion indeed reduced self-critical emotions among high identifiers. For low identifiers, we found either positive patterns or null effects. The effects we found on emotions also translated to perceptions of blame. High identifiers in the compassion condition blamed themselves less and the other person more, compared to high identifiers in the control condition. Compassion caused low identifiers to blame themselves more and the other person less, although these simple effects were weaker. These findings are particularly noteworthy given the subtle nature of the manipulation and the mechanism that we propose underlies the effects. To our knowledge, the present research is also the first to investigate this counterintuitive ‘other’ side to compassion in the interpersonal domain.

### Hypothesis 2: Perspective manipulation

We expected that when high identifiers got to write about the past event in the third- rather than first-person condition, this would reduce self-critical emotions and self-blame, and increase other-blame. Previous research has shown that a third-person perspective is associated with further subjective distance from an event, and in turn less affect and emotions [3,19–21]. Although in theory this could apply to both high- and low-identifiers, we particularly expected high identifiers to use this opportunity to distance themselves from the event, because they have a strong defensive motivation to do so. Research by Libby et al. [23] has shown that motivation may play a crucial role in predicting the effects of adopting a third-person perspective.

Our results support our prediction of negative effects of the third person perspective for high identifiers. However, we found consistent negative simple effect patterns for both high *and* low identifiers (and thus no significant overall interaction effects). The effects were strongest on guilt and regret and weakest on anger. Thus, the switch in perspective caused people to be less emotionally involved in general, regardless of their level of self-identification. This is interesting, because one could argue that the effect should be weaker for low self-identifiers, because they lack the defensive motivation to protect the past self. Instead, our findings on emotions are more consistent with a self-distancing account, which suggests that the third-person perspective should inherently arouse emotions to a lesser extent.

A possible explanation for this may relate to a distinction between different dimensions of perspective. The aforementioned research by Libby and colleagues [22, 23], which established the key role the self-concept relevant information can play in producing the effects of perspective, specifically concerned *visual* perspective. Manipulations of this dimension include explicit instructions to visualize a recalled event from a first-person perspective (e.g. to take the visual point of view one originally had during the experience) or third-person perspective (e.g. to take the visual point of view that an observer would have had during the recalled event) [22]. In contrast, in other lines of research manipulations are used in which participants are simply asked to *describe* events in the first-person (e.g. using the pronoun ‘I’) or the third-person (e.g. using the pronouns ‘he’ or ‘she’) [20]. The manipulation used in the present studies had more in common with the latter approach: although we did ask participants to not just describe but also recall the event from either a first-person or third-person perspective, we did not explicitly refer to their visual point of view and primarily emphasized their description of the event.

This distinction is important, because Libby and Eibach [22] argue that it is specifically the visual dimension of perspective that evokes the bottom-up (more concrete) and top-down (more abstract) representation of an event. In turn, they argue that it is this abstract construal of the event on which self-concept relevant information can exert a strong influence. Thus, one reason why we found unexpectedly similar effects for low and high identifiers in the present studies, might be because our manipulation focused more on the semantic, rather than visual dimension of perspective. It would be interesting to see whether a similar study to ours using a perspective manipulation aimed more explicitly at the visual dimension, would produce different effects.

However, we deem it unlikely that this distinction can fully account for our findings. Firstly, as mentioned above, we did also ask our participants to recall (not just write about) the event from the first- or third-person perspective. Secondly, even instructions that do purely target the language participants use to describe an event, can affect the visual representation of the situation to some extent. For example, Kross et al. [20] found that participants asked to write about a past event using third-person pronouns reported that they replayed the event in their mind from the perspective of an observer to a significantly larger degree than participants asked to write using first-person pronouns.

Most importantly, we *did* find differences between low and high identifiers on our measures of self-blame and other-blame (the overall interactions were nearly significant). Whereas the third-person perspective manipulation did not affect perceptions of blame among low identifiers, we found (in line with our predictions) that it reduced self-blame and increased victim-blame among high identifiers. The latter simple effects were both significant. Thus, although describing an event from a third-person perspective caused both high and low identifiers to feel less self-critical emotions, this effect only translated to perceptions of blame for high identifiers. This *does* seem to reflect the proposed motivational nature of the effect: only high self-identifiers have a need to reduce feelings of blame, and employ the increased distance inherent to the third-person perspective to do so. Future research may be able to shed more light on the reasons why the expected distinction between high and low identifiers did emerge on blame, but not on measures of self-critical emotions.

### Hypothesis 3: Salient difference manipulation

We asked participants either to list three ways in which they are different today compared to the past, or three ways in which they are similar. We predicted that high identifiers in the difference condition would show a reduction in self-critical emotions and self-blame, and an increase in other-blame. We expected that high identifiers would use this as an opportunity to shift the focus away from the past self.

We did not find support for this prediction. None of the simple effects for high identifiers were significant. For guilt, shame and regret the effect was even slightly positive, rather than negative, and the effect for other-blame was also slightly negative. However, the confidence intervals were so large (due to the fact that this meta-analysis was only based on two studies, and the effects in the two studies opposed each other) that these values really do not tell us much. For low identifiers, we expected a null effect or a positive effect, because these people already feel quite different to the person they were when the event took place. Our data supported the latter notion. It seems like the manipulation bolstered the already-existing mindset that they are currently different to the past self, and therefore made them even more open to self-critical emotions about their past behavior. They also blamed themselves more, and the other person less for the past event. So although we did not find a negative effect for high identifiers, the absence of a positive effect is still noteworthy, as this was found for low identifiers.

### Disidentifying

Finally, we conducted an exploratory analysis to see whether identification with the past self changed during the experiment as a result of the emotions that were evoked. To test this, we looked at the interaction effects between our pre-measure of identification and each of our self-critical emotions on our post-measure of identification. We did not formulate specific predictions. This is because in principle, high identifiers should be very unwilling to disengage from their past self. However, if other distancing strategies prove ineffective, it may be their only option.

We did not find significant overall interaction effects, but whereas we consistently found null effects for low identifiers, the simple effects for high identifiers were consistently negative. The interactions between pre-identification and guilt and regret were nearly significant, and the interactions with shame and anger were significant. In other words, the emotions that low identifiers reported feeling during the experiment did not lead to a change in the degree to which they identified with their past self at the end of the experiment. This makes sense, because these people already entered the experiment feeling quite different from the past self. For high identifiers, in contrast, we see that the emotions evoked during the experiment did lead to a decrease in identification by the end of the experiment. Thus, the more guilt, shame, anger and regret they reported feeling, the more they ended up distancing themselves from their past self. Or, framed differently, the more they managed to resist these emotions (for instance by means of the other three distancing mechanisms we studied), the more likely they were to maintain their high levels of identification.

It should be noted that the effect sizes we found in our analyses were small and that not all effects were significant. This is of course important to keep in mind when interpreting these results. In part, this might be explained by limitations to our method. One reason in particular could be that our instructions about the recalled event were not entirely clear. Although most participants gave answers that fit our instructions, there was a sizeable minority in each study whose descriptions were difficult to interpret. We applied conservative exclusion criteria, which could in part explain the weak effects.

### Self-identification vs. implicit theories

In the present research, we applied the logic from the intergroup domain to the question how individuals react to confrontations with past episodes where they harmed someone else in the interpersonal domain. We believe using the concept of identification, in this case with the past self rather than the group, is very helpful in this context because it can help us understand why some people may react very defensively to such a confrontation, whereas others are very open to it. An underlying assumption of the present research is not only that people differentiate between past and present selves (which is well-established in the literature), but that they identify with their past self to differing degrees. Our data support this idea. The weighted average of our pre-measure of past self-identification across all seven studies was 3.81, with a standard deviation of 1.67 (we used a seven-point scale). Scale scores ranged from one to seven in every single study. This indicates that people do identify with/feel similar to the person they were 5 to 10 years ago, and more importantly, that they do so to varying degrees.

One may wonder to what extent our concept of past self-identification differs from the more frequently studied implicit theories regarding personal attributes and feelings (e.g [2,3]). Although the two are likely related, we believe the latter are more a general set of beliefs about consistency and continuity, whereas self-identification is more directly self-relevant and emphasizes the relationship between the past and present selves more directly. To give an example, indicating that you have maintained the same values across time does not directly address the distinction between a past self and the present self. You might have maintained the same basic values, but still see yourself as a very different person compared to who you were five years ago. For instance, suppose a married man has always felt that one should not cheat on his partner, but ended up doing exactly that five years ago. We know that perceived failures can cause people to create shifts in identity, so let's suppose he now sees himself as a very different (much better, naturally) person than the person he was when he cheated on his partner five years ago. It seems very possible that this low past self-identifier would still indicate that he maintained the same values across time (and thus score high on a consistency item), because his belief that one should not cheat on his partner remains unchanged. Similarly, an example of a continuity belief might be the idea that there is a causal link between the different events that occur during one's life. Someone who is strongly identified with the person they used to be 5 years ago might agree with this, but so might the weakly self-identifying cheating man from the previous example. These examples just go to show that the two concepts certainly do not seem to fully overlap.

To test whether the two concepts are indeed distinct, we included a measure of self-continuity (adapted from [39]) in each of our studies. To see whether an analysis including self-continuity would produce different results, we also analyzed the two-way interaction between our compassion manipulation and our self-continuity measure. Although the patterns (especially for those high in self-continuity beliefs) were comparable to those for self-identification, we found no significant overall interaction effects and only one significant simple effect (on regret). This indicates that our self-identification measure had more explanatory power in this analysis. The overall correlation between our self-identification measure and self-continuity measure in the four studies that we used in this analysis was *r* = .53, 95% CI (.49, .58], Q(3) = 2.32, *p* = .508. This further demonstrates that the concepts are moderately associated, but do appear to be distinct.

### Conclusion

In the present research we applied concepts and findings derived from intergroup research to an interpersonal context in which a person has to recall a past event where they harmed someone else. Specifically, we investigated four potential ways in which people who are strongly identified with their past self can deal with a resulting identity threat: feeling compassion for the other person, describing the event from a third rather than first person perspective, emphasizing how they are different today compared to when the event took place, and disidentifying with their past self altogether.

It is important to note that we found the most consistent and strongest effects for the first strategy, feeling compassion, matching previous evidence in the intergroup realm [10]. This is noteworthy for a number of reasons: first of all, unlike the other strategies, feeling compassion has been specifically documented as having positive effects for interpersonal and intergroup relations. We did find these 'traditional' effects for people who are not strongly identified with the person they used to be, but found a decrease in self-critical emotions and self-blame, and an increase in victim blaming for highly self-identified people. Secondly, our compassion manipulation is arguably the most subtle and indirect of the three manipulations we included. Writing about the event from a third-person perspective and getting participants to emphasize differences both involve much more than a short instruction to think about the suffering of the other person and make the distance from the self clearer. It may be that one effective aspect of the compassion manipulation is that it is unconscious, in the sense that it involves some beneficial self-deception in which ego-defensive measures are not consciously considered [40]. Finally, and related to this, it might be the most realistic of our manipulations. It seems very plausible that in 'the real world', people are much more likely to experience a feeling of compassion when they recall these kinds of past events, than that they would naturally start thinking about the event from a third-person perspective, or work through a laundry list of ways in which they are a different person today prior to engaging with their memory of the event.

Ironically, it seems the most effective way to protect the self against a reminder of a past event that we might prefer to forget, is to have compassion for our victims. However, whether this strategy is adaptive in the long-term is debatable: consistent reductions in self-critical emotions could discourage us from repairing the harm we inflict, and thereby damage interpersonal relationships in the long run.

## Supporting information

S1 Appendix In the appendix, we investigate the role of severity of the events participants recalled. We report whether self-identification affected the severity of the recalled events, and re-ran our original analyses while controlling for severity (as well as the interaction between severity and each manipulation).
(DOCX)
