# Supplementary material for: Having pity on our victims to save ourselves: Compassion reduces self-critical emotions and self-blame about past harmful behavior among those who highly identify with their past self
Source: PLoS One. 2019 Dec 12;14(12):e0223945. doi: 10.1371/journal.pone.0223945 (PMC6907768; doi:10.1371/journal.pone.0223945)
Supplement: S1 Appendix — We report whether self-identification affected the severity of the recalled events, and re-ran our original analyses while controlling for severity (as well as the interaction between severity and each manipulation). (DOCX) [file pone.0223945.s001.docx]

**Appendix – The role of severity of the recalled event**

We assessed whether high and low self-identifiers recalled events that differed in their severity. It does not seem far-fetched that high self-identifiers may have strategically avoided recalling events that strongly implicate their past self, because they are still very much invested in seeing this past self in a positive light. Testing this is important, primarily because this could be a potential confound in our study, specifically for the compassion manipulation (as this was both the most novel, and the most subtle strategy we investigated).

To assess this, we asked two independent judges to analyse the event descriptions in all seven studies (1742 observations in total). For each description, the two judges were asked to indicate how severe they deemed the harm that was inflicted on a 7-point scale. Judges were instructed to refrain from providing a rating if they felt the description was not clear enough to accurately assess severity. As a result of this, 87 cases were omitted (12 in Study 1, 18 in Study 2, 12 in Study 3, 9 in Study 4, 8 in Study 5, 10 in Study 6, and 18 in Study 7). We calculated the intraclass correlation as a measure of interrater reliability. ICC(2,2) varied between .43 and .54. These values are classified as ‘fair’ by Cicchetti (1994).

**Effect of self-identification on severity of recalled event**

We first tested whether self-identification had an effect on severity. We therefore ran simple regressions in all seven studies with self-identification as the independent variable and severity of the recalled event as the dependent variable. We subsequently used D-STAT (Johnson, 1993) to convert the resulting t-values into r-values, and subsequently used MetaLight (Thomas et al., 2012) to calculate a meta-effect size.

The effect of self-identification on severity was significant, *r* = -.12, 95% CI [-0.17, -0.07], Q(3) = 4.30, *p*>.250. Thus, the more participants reported to identify with their past self, the lower was the severity of the event they recalled.

**Interaction between our manipulations and self-identification, after controlling for severity of recalled event**

On the basis of the aforementioned findings, it is clear that self-identification and severity are related. For the purposes of our study, however, the key question was whether this association explained the interaction effects that we reported in our study, particularly with regards to compassion. We also checked to what extent it could explain the interaction effects between identification and our perspective and salient difference manipulations.

We re-ran our original analyses, but this time statistically controlled for severity of the recalled event. We did so by adding severity as a covariate into our original analysis. To fully control for the potential influence of severity, we also calculated the interaction between severity and the manipulations, and included this interaction term as an additional covariate (Yzerbyt, Muller, & Judd, 2004). The outcomes of this analysis are presented in Table A (for the compassion manipulation), Table B (for the perspective manipulation), and Table C (for the salient difference manipulation).

With regards to compassion, the effect sizes of the overall interaction tended to be slightly lower than in our original analysis. An inspection of the simple effects revealed that this was primarily because the effects for low identifiers became weaker and less consistent, as can be seen in Figure A (see Fig 1 in the main text for a graphical comparison with the original analysis). However, it should be noted that the pattern of results for low identifiers was already quite inconsistent in our original analysis (as discussed in the main text). Crucially, the simple effects for high self-identifiers remained almost identical. Similarly, both the overall interaction effects and the simple effects did not significantly change in the analyses of the perspective (see Figure B) and salient difference manipulations (see Figure C) either.

Overall, these two analyses suggest that self-identification did influence the severity of the events that were recalled, but that it could not account for the interaction effects we found with compassion (particularly for high identifiers), and our perspective and salient difference manipulations. One could argue that the main effect on severity points to an additional ‘defense mechanism’, in addition to the distancing strategies we investigated. High self-identifiers may have used the free recall procedure we used in our studies as their first line of defense: because they are still strongly identified with the person they were 5 to 10 years ago, they may have purposely recalled less severe events. However, it is of course also possible that low self-identifiers are not strongly identified with their past selves for the very reason that they *have* experienced more severe events: they may have created a shift in their identity in order to cope with that (Wilson & Ross, 2003). As our analysis is of a correlational nature, we cannot establish the direction of this effect on the basis of our data – this is something that could be investigated in future research.

**Table A**

| **DV** | **Overall interaction** | **Simple effect  Low Identifiers** | **Simple Effect High Identifiers** |
| --- | --- | --- | --- |
|  |  |  |  |
| Guilt | -.06 [-.12, .00]  Q(3) = 2.10, *p*>.552 | .03 [-.05, .11]  Q(3) = 4.49, *p=*.213 | -.05 [-.12, .01]  Q(3) = 3.40, *p*>.250 |
| Shame | -.06 [-.13, .00]  Q(3) = 2.40, *p*>.250 | .03 [-.05, .11]  Q(3) = 4.24, *p=*.237 | -.06 [-.12, .01]  Q(3) = 2.84, *p*>.250 |
| Anger | -.04 [-.10, .03]  Q(3) = 1.89, *p*>.250 | .03 [-.04, .09]  Q(3) = 1.84, *p>*.250 | -.03 [-.09, .04]  Q(3) = 2.77, *p*>.250 |
| Regret | -.05 [-.11, .02]  Q(3) = 0.84, *p*>.250 | -.01 [-.09, .07]  Q(3) = 4.18, *p=*.243 | -.08 [-.15, .00]  Q(3) = 3.93, *p>*.250 |
| Self-blame | -.07 [-.14, .00]  Q(3) = 3.52, *p*>.250 | .01 [-.10, .13]  Q(3) = 9.51, *p=*.02 | -.07 [-.13, .-01]  Q(3) = 2.77, *p*>.250 |
| Other-blame | -.07 [-.00, .14]  Q(3) = 3.62, *p*>.250 | -.03 [-.12, .06]  Q(3) = 5.66, *p=*.130 | .07 [.00, .13]  Q(3) = 1.69, *p*>.250 |

Meta-effect sizes for the interaction between the **compassion manipulation** and self-identification, after controlling for severity of the recalled event. We present r-values along with a 95% CI, as well as the Q-statistic, for both the overall interaction effects and the simple effects for low and high identifiers.


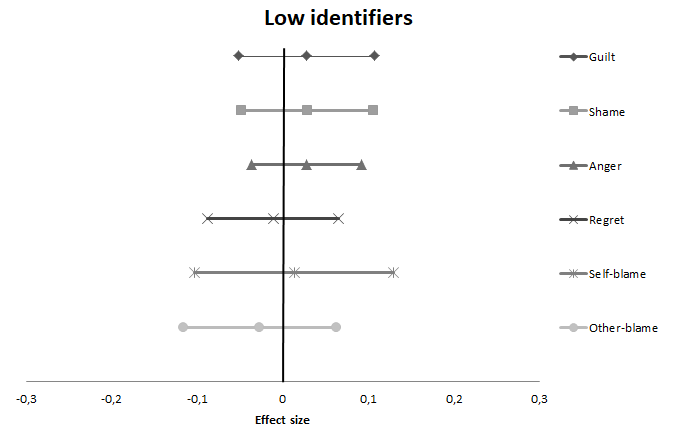

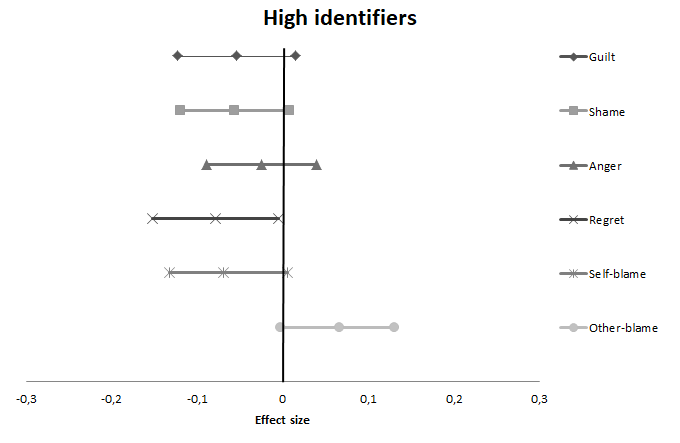


**Figure A***.* A forest plot showing the overall meta-analytic simple main effects of our **compassion manipulation** on the dependent variables (based on four studies), *after controlling for the severity of the event participants recalled (as coded by 2 independent judges).* Effect sizes are provided along with their confidence intervals. A positive value indicates that participants in the compassion condition scored higher than participants in the control condition, a negative value that they scored lower.

**Table B**

| **DV** | **Overall interaction** | **Simple effect  Low Identifiers** | **Simple Effect High Identifiers** |
| --- | --- | --- | --- |
|  |  |  |  |
| Guilt | .02 [-.07, .01]  Q(3) = 2.85, *p*=.241 | -.07 [-.14, .00]  Q(3) = 1.11, *p>*.250 | -.05 [-.13, .02]  Q(3) = 2.09, *p*>.250 |
| Shame | .03 [-.06, .12]  Q(3) = 2.76, *p*>.250 | -.05 [-.12, .03]  Q(3) = 1.49, *p>*.250 | -.01 [-.08, .07]  Q(3) = 1.39, *p*>.250 |
| Anger | .02 [-.07, .11]  Q(3) = 2.92, *p=*.232 | -.02 [-.09, .06]  Q(3) = 1.70, *p>*.250 | -.00 [-.07, .08]  Q(3) = 1.39, *p*>.250 |
| Regret | -.00 [-.09, .08]  Q(3) = 2.79, *p=*.248 | -.06 [-.14, .01]  Q(3) = 1.35, *p>*.250 | -.07 [-.15, .00]  Q(3) = 1.51, *p>*.250 |
| Self-blame | -.06 [-.16, .04]  Q(3) = 3.41, *p=*.182 | .01 [-.12, .14]  Q(3) = 5.65, *p=*.059 | -.07 [-.15, .00]  Q(3) = 1.08, *p*>.250 |
| Other-blame | .07 [-.01, .14]  Q(3) = 2.00, *p*>.250 | -.02 [-.18, .16]  Q(3) = 9.91, *p=*.007 | .09 [.01, .16]  Q(3) = 1.31, *p*>.250 |

Meta-effect sizes for the interaction between the **perspective manipulation** and self-identification, after controlling for severity of the recalled event. We present r-values along with a 95% CI, as well as the Q-statistic, for both the overall interaction effects and the simple effects for low and high identifiers.


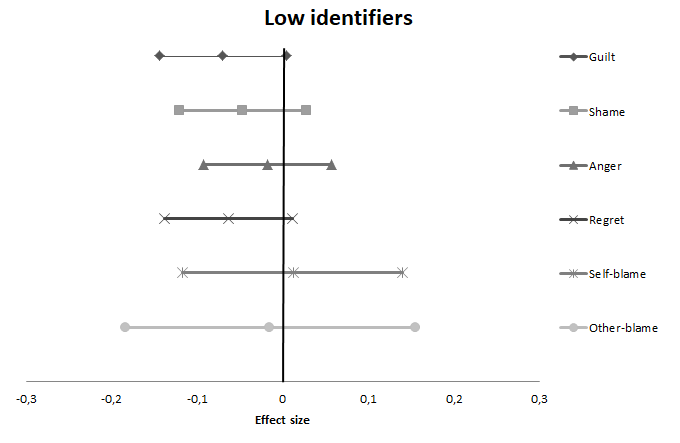

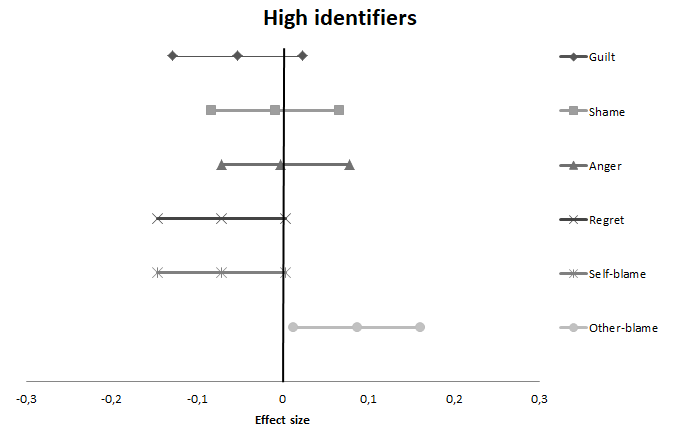


**Figure B***.* A forest plot showing the overall meta-analytic simple main effects of our **perspective manipulation** on the dependent variables (based on four studies), *after controlling for the severity of the event participants recalled (as coded by 2 independent judges).* Effect sizes are provided along with their confidence intervals. A positive value indicates that participants in the third person-perspective condition scored higher than participants in the first person-perspective condition, a negative value that they scored lower.

**Table C**

| **DV** | **Overall interaction** | **Simple effect  Low Identifiers** | **Simple Effect High Identifiers** |
| --- | --- | --- | --- |
|  |  |  |  |
| Guilt | -.05 [-.16, .07]  Q(3) = 1.79, *p*=.160 | .10 [.02, .19]  Q(3) = 0.00, *p>*.250 | .04 [-.13, .21]  Q(3) = 4.07, *p=*.044 |
| Shame | -.06 [-.21, .09]  Q(3) = 3.23, *p=*.072 | .12 [.03, .20]  Q(3) = 0.40, *p>*.250 | .03 [-.13, .19]  Q(3) = 3.71, *p=*.054 |
| Anger | -.10 [-.26, .06]  Q(3) = 3.82, *p=*.051 | .13 [.05, .21]  Q(3) = 0.00, *p>*.250 | -.02 [-.25, .21]  Q(3) = 7.59, *p=*.006 |
| Regret | -.08 [-.16, .00]  Q(3) = 0.94, *p>*.250 | .15 [.07, .23]  Q(3) = 0.05, *p>*.250 | .04 [-.06, .14]  Q(3) = 1.33, *p=*.249 |
| Self-blame | -.07 [-.17, .03]  Q(3) = 1.41, *p=*.235 | .09 [.01, .17]  Q(3) = 0.29, *p>*.250 | -.01 [-.11, .08]  Q(3) = 1.35, *p=*.246 |
| Other-blame | .05 [-.04, .13]  Q(3) = 0.12, *p*>.250 | -.09 [-.17, -.01]  Q(3) = 0.15, *p>*.250 | -.03 [-.11, .06]  Q(3) = 0.80, *p*>.250 |

Meta-effect sizes for the interaction between the **salient difference manipulation** and self-identification, after controlling for severity of the recalled event. We present r-values along with a 95% CI, as well as the Q-statistic, for both the overall interaction effects and the simple effects for low and high identifiers.


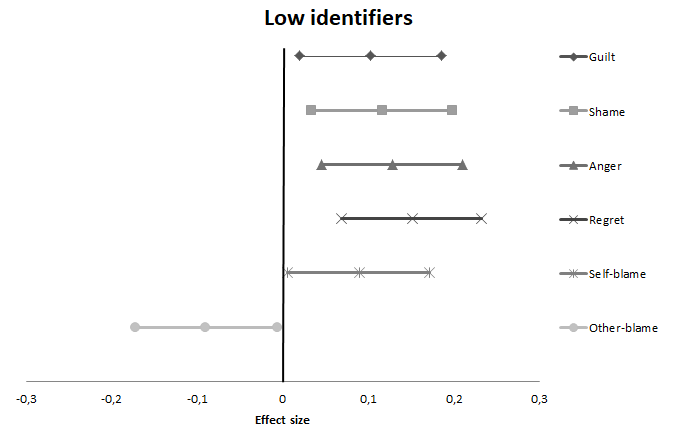

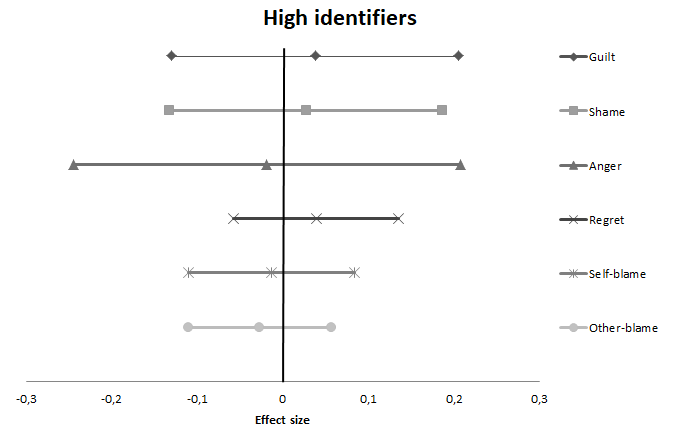


**Figure C***.* A forest plot showing the overall meta-analytic simple main effects of our **salient difference manipulation** on the dependent variables (based on four studies), *after controlling for the severity of the event participants recalled (as coded by 2 independent judges).* Effect sizes are provided along with their confidence intervals. A positive value indicates that participants in the difference-focused condition scored higher than participants in the similarities-focused condition, a negative value that they scored lower.
